# Beauty in artistic expressions through the eyes of networks and physics

**DOI:** 10.1098/rsif.2019.0686

**Published:** 2020-03-11

**Authors:** Matjaž Perc

**Affiliations:** 1 Faculty of Natural Sciences and Mathematics, University of Maribor, Koroška cesta 160, 2000 Maribor, Slovenia; 2 Department of Medical Research, China Medical University Hospital, China Medical University, Taichung, Taiwan; 3 Complexity Science Hub Vienna, Josefstädterstraße 39, 1080 Vienna, Austria

**Keywords:** complexity, entropy, network science, data science, self-organization

## Abstract

Beauty is subjective, and as such it, of course, cannot be defined in absolute terms. But we all know or feel when something is beautiful to us personally. And in such instances, methods of statistical physics and network science can be used to quantify and to better understand what it is that evokes that pleasant feeling, be it when reading a book or looking at a painting. Indeed, recent large-scale explorations of digital data have lifted the veil on many aspects of our artistic expressions that would remain forever hidden in smaller samples. From the determination of complexity and entropy of art paintings to the creation of the flavour network and the principles of food pairing, fascinating research at the interface of art, physics and network science abounds. We here review the existing literature, focusing in particular on culinary, visual, musical and literary arts. We also touch upon cultural history and culturomics, as well as on the connections between physics and the social sciences in general. The review shows that the synergies between these fields yield highly entertaining results that can often be enjoyed by layman and experts alike. In addition to its wider appeal, the reviewed research also has many applications, ranging from improved recommendation to the detection of plagiarism.

## 1. Introduction

The past decade has seen data science emerge as the new buzzword in the world of research. In 2012, Thomas H. Davenport and D. J. Patil at *Harvard Business Review* called being a data scientist ‘the sexiest job of the 21st century’. And although some argue that data science is not much more than classical statistics with a touch of modern flair, the richness of digital data today, in terms of volume, diversity and speed of change [1], requires synergies that transcend disciplinary boundaries. Data science uses methods and techniques from mathematics, physics, computer science, information science and of course statistics to make sense of the vast amounts of digital data that are daily added to computer servers worldwide. Visualization wizardry is likewise key for the data science to shine through, as opposed to rather one-dimensional and often dull representations one often finds in hardcore statistics books.

And it is this deluge of digital data that is often the bridge between the social and natural sciences, and, as we will review in what follows, also between art and natural sciences, and between art and physics and network science in particular. One may wonder whether classical physics has anything to do with societal challenges and with modern human societies. Yet research has shown that collectively we often behave no differently than particles in matter [2]. Not exactly, of course, but close enough for methods of statistical physics to be applied prolifically to subjects such as traffic [3], crime [4], epidemics [5], vaccination [6], cooperation [7], climate inaction [8], as well as antibiotic overuse [9] and moral behaviour [10]. The physics of social systems, or social physics [11] or sociophysics [12]—regardless of the name tag—has had an excellent growth pattern over the past couple of decades, spurred on by advances in statistical and computational physics, the availability of data, and the coming of age of related fields of research such as network science [13–16] and computational social science [17].

Looking at this development historically, it is, in fact, safe to claim that synergies between physical and social sciences have been around for centuries. Surely not as precise and useful as they are now, but nevertheless. Already in the seventeenth century, Thomas Hobbes based his theory of the state on the laws of motion, in particular, on the principle of inertia, which was then deduced by his contemporary Galileo Galilei. The ‘invisible hand’ proposed by Adam Smith in the second half of the eighteenth century is also eerily similar to the now famous notions of economic and social self-organization [18,19], and at the time was deemed to be as dependable in operation as the law of gravity [20]. And in the nineteenth century, the evolving physical theories of matter as a vast collection of atoms and molecules inspired a statistical view of societies and the predictable averages therein. French political and economic theorist Henri de Saint-Simon indeed proposed that society could be described by laws similar to those in physics. Just as the random movements of molecules in a gas yield the mathematically simple gas laws, it was fathomed that so might societies be predictable in the collective. Thus, as Philip Ball argued aptly [21], early sociology might have been constructed according to an unspoken faith that there was a kind of ‘physics of society’ that exists but is as yet somewhat in the stars.

But it is one thing to investigate and mathematically describe measurable phenomena in human societies, and a whole other to try and do the same for art. There is definitively something to art that will remain forever untouchable to the coldness of measure and science. But in spite of knowing full well we will likely never be able to understand it all, many have tried and succeeded in bridging the divide, with often deeply satisfying outcomes for both science and art. An example of this line of research is computational aesthetics [22], having roots already in the first half of the twentieth century, when the American mathematician George D. Birkhoff proposed the ratio between order and complexity as an aesthetic measure [23]. In fact, the key mission of computational aesthetics is to develop scientific means to quantify beauty and model human aesthetic perception [24], although it influences also computer-generated art.

**Figure 1.**
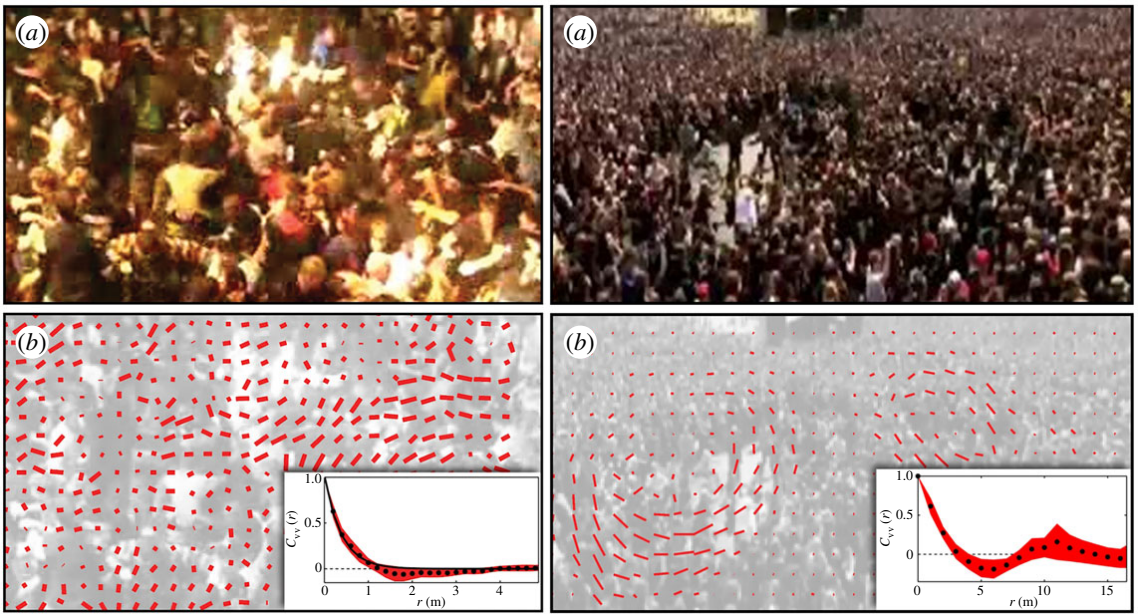
Collective human motion at heavy metal concerts. Left a mosh pit, akin to a disordered gas-like state. Right a circle pit, akin to an ordered vortex-like state. Both mosh and circle pits can be reproduced in flocking simulations (bottom row), demonstrating that human collective behaviour is consistent with the predictions of simplified models. For details concerning the mathematical model and the interpretation of insets in the bottom row with refer to the original work. Reproduced from [25].

As something of a middle ground, one can come across research on human collective behaviour that is due to art, more specifically due to heavy metal music. Silverberg *et al.* [25] studied the collective motion of humans at heavy metal concerts, showing that such social gatherings generate extreme behaviours, ranging from a disordered gas-like state to an ordered vortex-like state, as shown in figure 1. The differences between such so-called mosh and circle pits can be reproduced in flocking simulations, thus confirming that even relatively very simple mathematical models can accurately describe the essence of human collective behaviour.

In what follows, we review research dedicated to culinary arts, visual arts and musical arts, and where appropriate we also touch upon cultural history [26,27] and culturomics [28], and we describe the connections between physics and the social sciences. We conclude with a discussion of the reviewed research and an outlook for future research.

## 2. Culinary arts

It is somewhat debatable whether cooking is art. Putting together a hamburger at a McDonald’s is more automation than anything, and it is certainly no art. But creating a delicious meal for your loved ones can be art, and culinary arts, in particular, have to do with the preparation and cooking of foods. But what does this have to do with physics and networks?

**Figure 2.**
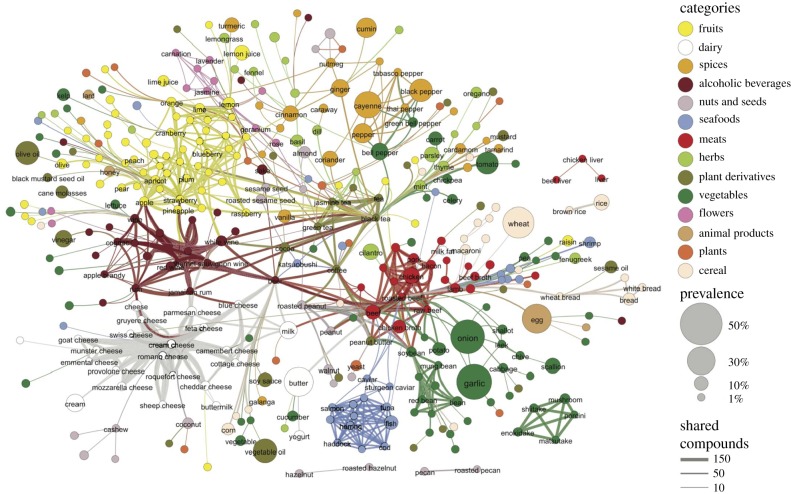
The flavour network. Each node represents an ingredient, the colour of nodes indicates food categories listed top right, and the node size, as show bottom right, reflects the prevalence of an ingredient in recipes. Two ingredients are connected if they share a significant number of flavour compounds, while the thickness of links represents the number of shared compounds between the two ingredients. Only statistically significant links at *p*-value cut-off 0.04 are show as otherwise the network would be too dense. For further details, we refer to the original work. Reproduced from [29].

Perhaps not much on first glance, yet this is deceiving. About eight years ago Ahn *et al.* [29] published a paper where they introduced the flavour network to uncover fundamental principles of food pairing. The backbone of the flavour network is shown in figure 2. They have argued that, given the increasing availability of information on food preparation, such as at cookpad.com and foodpairing.com, a data-driven approach can open up new avenues towards a systematic understanding of culinary art. Their research showed that North American and Western European cuisines have a statistically significant tendency towards sharing flavour compounds in recipe ingredients. By contrast, East Asian and Southern European recipes are much less likely to have ingredients that share flavour compounds. And vice versa, the more compounds are shared by two ingredients, the more likely they appear together in North American recipes, and less likely to appear together in East Asian cuisine. In exploring the mechanism responsible for these differences, Ahn *et al.* [29] found that the food-pairing effect is due to only a couple of ingredients that are frequently used in a particular cuisine. This would be milk, butter, cocoa, vanilla, cream and egg in North America, and beef, ginger, pork, cayenne, chicken and onion in East Asia, for example. These findings resonates with the well-known ‘flavour principle’ [30], according to which regional cuisines are due to just a few key ingredients, such as soy sauce for Asia or paprika and onion for Hungary.

However, already prior to the flavour network study [29], Kinouchi *et al.* [31] studied the statistics of ingredients and recipes taken from the Brazilian *Dona Benta*, the British *New Penguin Cookery Book*, the French *Larousse Gastronomique* and the medieval *Pleyn Delit*. They have observed universal distributions of ingredients with scale-invariant behaviour, which motivated a mathematical model akin to growth and preferential attachment in networks [32], or more generally to the Matthew effect [33]. The so-called copy-mutate model of culinary evolution was shown to fit the empirical data well. The authors also argued that the model indicates an evolutionary dynamics governing the evolution of recipes over the centuries where idiosyncratic ingredients are preserved in a manner similar to the founder effect in biology [34].

In more recent years, several research works followed up to study culinary arts with methods of network science, physics and related approaches. For example, Teng *et al.* [35] showed how to improve recipe recommendations based on ingredient networks. Their research showed that recipe ratings can be predicted well with features from ingredient networks and nutritional information. The food-bridging hypothesis was also proposed, assuming that if two ingredients do not share a strong molecular or empirical affinity, they may become affine through a chain of pairwise affinities [36]. Together with the food-pairing hypothesis [29], four classes of cuisine can then be distinguished. Namely, East Asian cuisine that tends to avoid food-pairing and food-bridging, Latin American cuisine that tends to embrace food-pairing and food-bridging, Southeastern Asian cuisine that tends to avoid food-pairing but embraces food-bridging and Western cuisine that embraces food-pairing but avoids food-bridging [36].

Relatively, smaller-scale research efforts have also been made, for example, looking specifically at Arab cuisine and whether it abides to the food-pairing hypothesis [37]. Flavour pairing was also studied for the medieval European cuisine [38], where authors investigated the flavour pairing hypothesis historically, focusing in particular also on the role of data incompleteness and error. To that effect, two separate chemical compound datasets with different levels of cleanliness were used, showing that they give conflicting conclusions with regards to the flavour pairing hypothesis in medieval Europe. Possible inferences about the evolution of culinary arts when many new ingredients are suddenly made available were also presented and discussed. The geography and similarity of regional cuisines in China has also been studied [39]. Research showed that geographical proximity rather than climate similarity is the crucial factor that determines the similarity of regional cuisines in China. Following the Kinouchi *et al.* [31] copy-mutate model of culinary evolution, similar models have also been proposed specifically for different Indian cuisines [40]. Authors argued that, in addition to the identified similarities and differences between the regions, a comparison of these models at the level of flavour compounds might open up a path towards molecular level studies that would associate specific ingredients with non-communicable diseases such as diabetes.

Apart from geographical interests and periodization, research at the interface of culinary arts and the more harder sciences also produced gems such as ‘Tell me what you eat, and I will tell you where you come from: A data science approach for global recipe data on the web’ [41] where the message is squarely in the title. It also gave rise to food computing [42], which acquires and analyses heterogeneous food data from disparate sources for perception, recognition, retrieval, recommendation and monitoring of food. Computational approaches can then be applied to address food-related issues in medicine, biology, gastronomy and agronomy. Online food preferences have also been explored based on server log data from a large recipe platform on the web [43]. Research revealed that recipe preferences are partly driven by ingredients, that recipe preference distributions exhibit more regional differences than ingredient preference distributions, and that weekday preferences are clearly distinct from weekend preferences. Along similar lines, food consumption and dietary patterns were also studied through twitter [44], aptly concluding ‘you tweet what you eat’, as well as through web usage logs [45].

We conclude this section with a reference to a contemporary book titled *Everyone Eats* [46], which explores why we eat what we eat, why some love spices, sweets and coffee, and why rice become such a staple food throughout so much of eastern Asia. With a focus on the social and cultural reasons for our food choices, the book may be a nice companion for further exploring this fascinating subject beyond physics and networks.

## 3. Visual arts

Patterns in nature are common and often beautiful and intriguing [47]. Pattern formation is thus, expectedly, also common in physics, chemistry and biology research, where emergent ordered and disordered structures often require quantification [48–54]. Indeed, the subject has been vibrant and popular ever since the seminal research by Alan Turing on the chemical basis of morphogenesis [55]. Perhaps thus not surprisingly, many methods that are often used in physics to quantify and study patterns can also be used to study art with hardly any modification.

The challenge up until recently was how to get visual art into quality digital form, and how to do so for a large number of artworks. With wikiart.org this challenge has been beautifully resolved, which opened up the path to large-scale analysis with methods of physics. Using these data, Sigaki *et al.* [56] studied the history of art paintings through the lens of entropy and complexity. Almost 140 thousand paintings, spanning nearly a millennium of art history, have been included in the research. Interestingly, it was shown that the complexity–entropy plane reflects traditional concepts in art history, such as Wölfflin’s dual concepts of linear versus painterly and Riegl’s dichotomy of haptic versus optic [57,58]. Linear artworks are composed of clear and outlined shapes, while painterly artworks have contours that are subtle and smudged for merging image parts and passing the idea of fluidity. Similarly, haptic artworks depict objects as tangible discrete entities, isolated and circumscribed, whereas optic artworks represent objects as interrelated in deep space by exploiting light, colour and shadow effects to create the idea of an open spatial continuum. Relating this to complexity and entropy, it was shown that linear/haptic artworks are described by small values of entropy *H* and large values of complexity *C*, while painterly/optic artworks are expected to yield larger values of *H* and smaller values of *C*.

**Figure 3.**
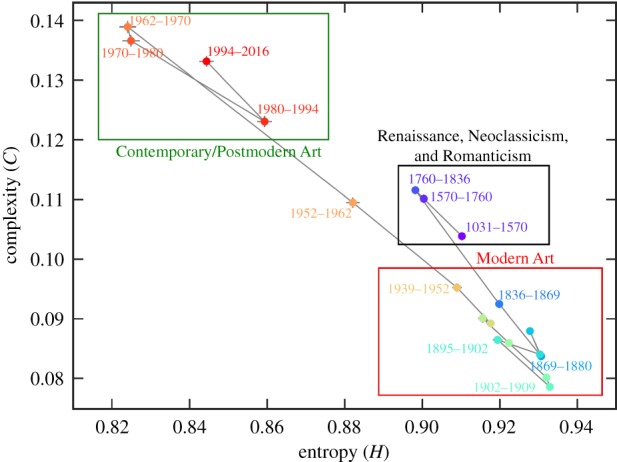
Quantifying the evolution of artworks through the history of art. Depicted is the temporal evolution of the average values of permutation entropy *H* and statistical complexity *C*. Each dot in the complexity–entropy plane corresponds to the average values of *H* and *C* for a given time interval, as indicated in the plot. Error bars represent the standard error of the mean. The highlighted regions show different art periods (black: Renaissance, Neoclassicism and Romanticism; red: Modern Art; green: Contemporary/Postmodern Art). We observe that the complexity–entropy plane correctly identifies different art periods and the transitions among them. Reproduced from [56].

These facts then allowed Sigaki *et al.* [56] to quantify the evolution of artworks through the history of art. To do so, the average values of *H* and *C* after grouping the images by date were computed. Results shown in figure 3 reveal that the artworks produced between the ninth and the seventeenth century are on average more regular/ordered than those created between the nineteenth and the mid-twentieth century. Also, the artworks produced after 1950 are even more regular/ordered than those from the two earlier periods. It can be observed further that the pace of changes in the complexity-entropy plane intensifies after the nineteenth century. Notably, this period indeed coincides with the emergence of several artistic styles such as Neoclassicism and Impressionism. Moreover, the three outlined regions correspond well with the main divisions of art history, as indicated in figure 3.

**Figure 4.**
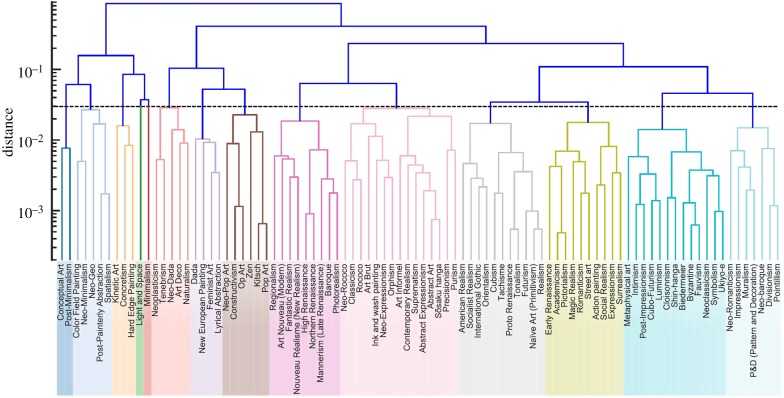
Hierarchical organization of artistic styles. Dendrogram representation of the distance matrix obtained by applying the minimum variance method. The 14 groups of styles indicated by the colourful branches are obtained by cutting the dendrogram at the threshold distance 0.03. This value maximizes the silhouette coefficient, and it is thus a natural number for defining the number of clusters in the dataset. For further details, we refer to the original work. Reproduced from [56].

Going a step further, complexity and entropy can also be used to distinguish different artistic styles in the *H* − *C* plane [56]. Since the values of *H* and *C* capture the degree of similarity among artistic styles regarding the local ordering of image pixels, a test for a possible hierarchical organization of styles with respect to this local ordering is thus possible. To do so, the Euclidean distance between a pair of styles in the complexity–entropy plane can be considered as a dissimilarity measure between them. Thus, the closer the distance between two artistic styles, the more significant their similarity, and vice versa. Results obtained with this procedure are shown in figure 4, which can be interpreted as a tree of art, akin to the tree of life in biology, as per the metaphor made famous by Charles Darwin in his book *On the Origin of Species*.

**Figure 5.**
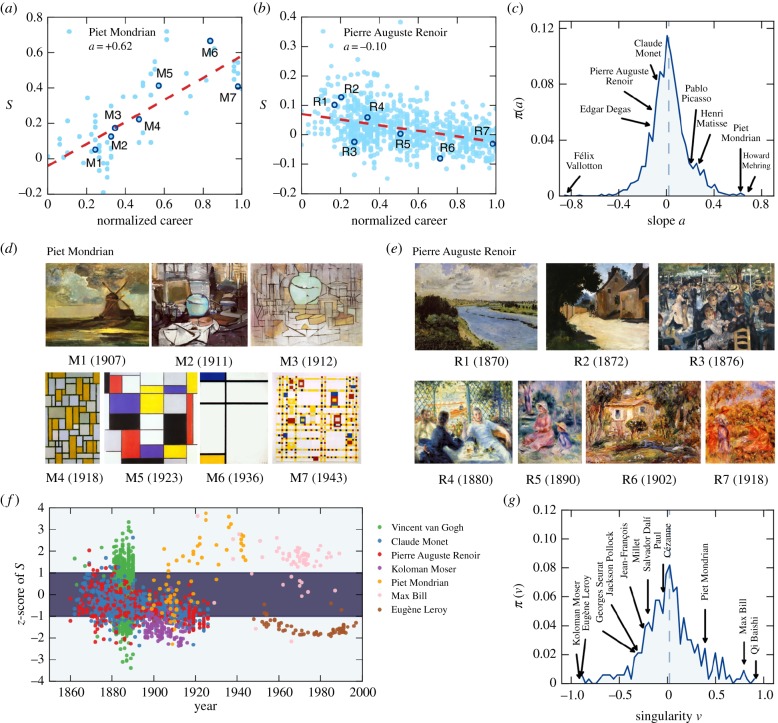
An in-depth analysis of individual painters. (*a*,*b*) The growth of the seamlessness in the paintings of Mondrian and Renoir over their career. Dashed red lines indicate best linear fits. In (*c*), a histogram of these linear fits is shown for 1326 artists that produced paintings in at least five distinct years. A few notable artists are indicated. (*d*,*e*) Samples of paintings by Mondrian and Renoir that highlight the stylistic changes of the two artists over their careers. (*f*) The singularity of paintings by seven select artists, while (*g*) shows the histogram of the singularity of 330 artists with more than 40 paintings. For further details, we refer to the original work. Reproduced from [60].

Of course, the study by Sigaki *et al.* [56] is neither the first nor the last along this line of research. Already four years earlier Kim *et al.* [59] conducted a large-scale quantitative analysis of painting arts with the aim to bridge the gap between art and science, focusing on the usage of individual colours, the variety of colours, and the roughness of the brightness. Their research revealed a difference in colour usage between classical paintings and photographs, and a significantly lower colour variety during the medieval period. An increment in the roughness exponent in painting techniques such as chiaroscuro and sfumato has also been reported, consistent with the historical circumstances of the period. The same group later also studied the heterogeneity in chromatic distances in images of the modern era [60], where they noted rightfully that aggregate statistics are a poor measure for the individuality of painters since the differences can stem from different painters, or from the same painters simply employing different styles. An insightful analysis from [60] is shown in figure 5, where representative individual painters are characterized in detail. Two distinct yet complementary aspects of stylistic individuality are considered, namely the evolution over their careers, and uniqueness relative to the dominant style of the period. Ultimately, this research provides a treasure of insights for an extraordinary expansion in creative diversity and individuality that defines the modern era.

In addition to the above-reviewed large-scale research attempts at quantifying visual art, it is important to note that the idea itself can be traced as far back as 1933, when the American mathematician George D. Birkhoff published his book *Aesthetic Measure* [23], which also gave rise to computational aesthetics [22]. Therein, he proposes to use the ratio between the number of regularities found in an image and the number of elements in an image as a quantitative aesthetic measure. But first applications happened only at the turn of the twenty-first century, where the work of Taylor *et al.* [61] showed that Pollock’s paintings are characterized by an increasing fractal dimension over the course of his artistic career. This subsequently inspired the application of fractal analysis to the authenticity of paintings [62–66], to the evolution of artists [67,68], to the statistical properties of paintings [69] and artists [70–72], to art movements [73] and to other visual expressions [74–76]. And we would be remiss not to mention that fractal art is a fascinating subject on its own right [77]. It is common in Islamic culture, for example on the main dome of the Selimiye Mosque in Turkey, and in Hindu Temples around the world. There is also innovative recent research focusing on the analysis of large-scale datasets of paintings and other visual art expressions by means of the estimation of their average brightness and saturation [78,79].

To end the section on visual arts, we note that in addition to art paintings, sculpture, ceramics, photography, video, filmmaking, design, crafts and architecture of course also fall under this category. Notably, filmmaking has been studied in terms of actor networks—where two actors are connected if they appeared together in a movie. One of the more recent attempts in this direction is the social network analysis of character interactions in the Stargate and Star Trek television series [80]. Research revealed that the character networks of both series have small-world properties, and that the underlying network structure of an episode can tell us something about the complexity of the storyline in that particular episode. Episode networks were found to be either closed networks, networks with bottlenecks that connect otherwise disconnected clusters, or a mixture of the two, which could be linked to the corresponding storylines upon a more detailed reading. But other forms of visual arts have yet to receive their link with physics and networks.

## 4. Musical arts

Research linking musical arts with science has the longest tradition amongst the arts considered in this review. Accordingly, we focus only on a selection of relatively recent research efforts that build predominantly on physics and network science as the bridges between the two disciplines.

We begin with Park *et al.* [81], who studied the topology and evolution of the network of western classical music composers. Based on the data from arkivmusic.com and allmusic.com, a bipartite network consisting of CDs and composers as the two node classes was created. Specifically, an edge between two nodes was drawn when a composer’s pieces was recorded on the CD. A single-mode projection of just composers was also made, where two composers were connected if they have been co-featured on a CD. Based on this, Park *et al.* [81] reported a wealth of results, including that the networks exhibit characteristics common to many real-world networks, such as the small-world property, the existence of a giant component, high clustering and heavy-tailed degree distributions. They have also explored the global association patterns of composers via centrality, assortative mixing and community structure, which suggest an intriguing interplay between the networks of musicians and our musicological understanding of the western musical tradition. Moreover, the study of the growth of the CD-composer bipartite network over time revealed superlinear preferential attachment as a strong candidate for explaining the increasing concentration of edges around top-degree nodes and the power-law degree distributions.

**Figure 6.**
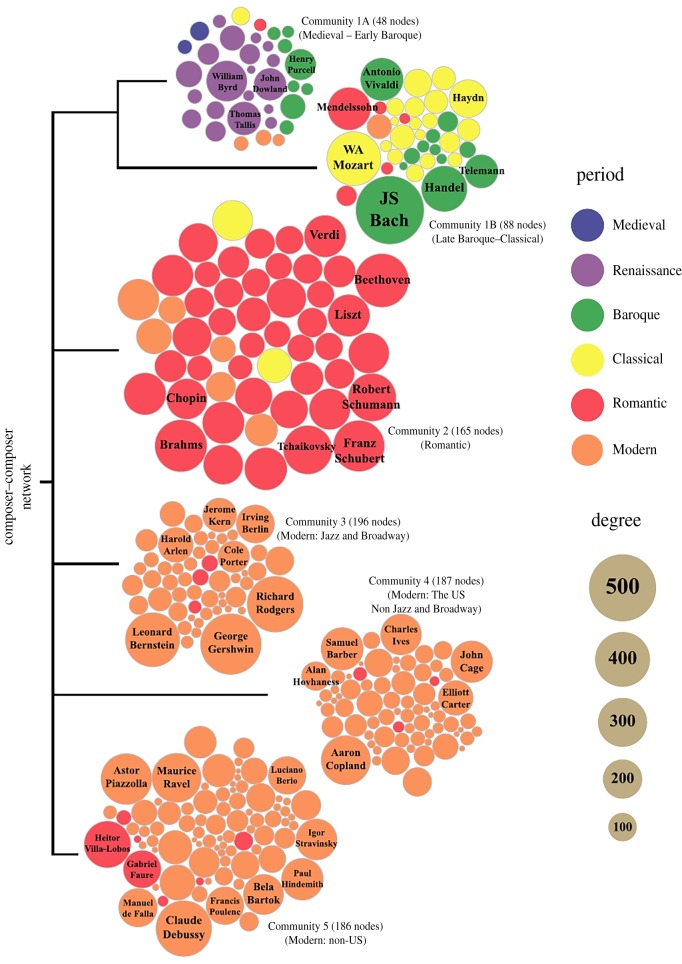
The community structure of the composer–composer network. Depicted are the five largest composer communities, which cover 6.2% of the composers who account for 60.1% of degrees. The communities correspond well to the established period definitions in classical music history. On the right is the colour code for the periods that are represented in each community, and the scale for their size. For details, we refer to the main text and the original work. Reproduced from [81].

From this, we review results concerning the community structure, as shown in figure 6. Six sizable communities are depicted, which account for 99% of the 878 nodes with known periods. Fascinatingly, the communities roughly correspond to the different musical periods, such as Renaissance and early Baroque (1A), late Baroque and Classical (1B), Romantic (2) and Modern (remaining three communities). The original work also lists notable composers in each of these communities, thus showcasing beautifully the power of network science to explore art in insightful ways.

**Figure 7.**
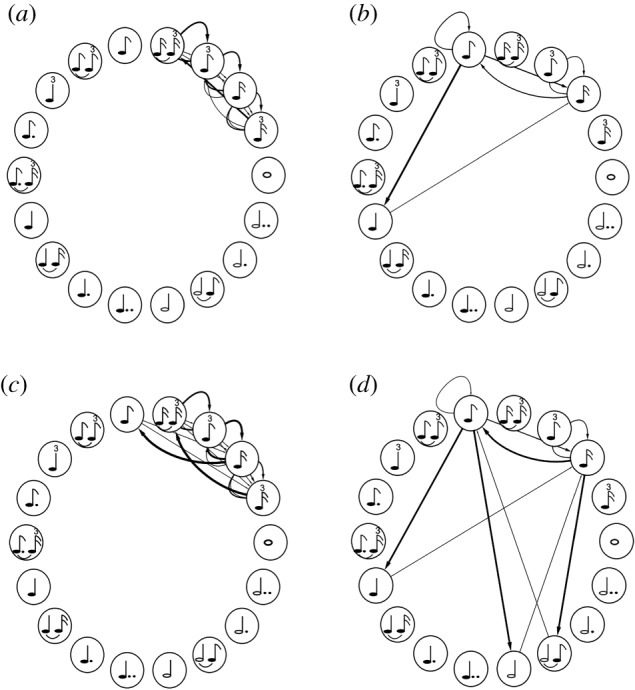
Digraph examples of four music samples as studied in [82]. Depicted are *How Blue Can You Get* by B. B. King (*a*), *Fotografia* by Tom Jobim (*b*), *Is This Love* by Bob Marley (*c*) and *From Me to You* by The Beatles (*d*). Reproduced from [82].

A relatively recent classic in terms of a more physics-inspired approach, although still based on networks, is the paper by Corrêa *et al.* [82], where authors explore the problem of automatic music genre classification by exploring rhythm-based features from a complex network representation. Specifically, a Markov model is proposed to analyse the temporal sequence of rhythmic notation events, and a feature analysis is performed by using the principal components analysis and the linear discriminant analysis. The former is an unsupervised multivariate statistical approach, while the later is a supervised one. Two classifiers are also applied to identify the category of rhythms, namely the parametric Bayesian classifier under the Gaussian hypothesis and agglomerative hierarchical clustering. Figure 7 shows how the digraphs used for the analysis were created. It can be observed that only the durations of the notes, respecting the sequence in which they occur in the sample, were used to create the digraphs. Each vertex of the digraph represents one possible rhythm notation, such as a quarter note, a half note, an eighth note, and so on. The edges reflect the subsequent pairs of notes. For example, if there is an edge from vertex *i*, represented by a quarter note, to a vertex*j*, represented by an eighth note, this means that a quarter note was followed by an eighth note at least once. Moreover, the thicker the edges, the stronger the link between these two nodes.

Based on this approach, Corrêa *et al.* [82] have shown that the musical rhythms are surprisingly complex and contain many redundancies, and that many features are required to separate them, in fact too many to review here (please see original work). Research also revealed that by allowing multi-genre classification a generalization of the genre taxonomy can be achieved since new sub-genres emerge spontaneously from the original genres. Although the study focused only on the rhythmic analysis, authors noted that a deeper analysis of the rhythms can be performed to enhance the effectiveness of the methodology.

More recently, partly the same group of authors studied whether ten compositions by the Baroque composer Johann Sebastian Bach arose from a Markov chain [83], by using the recurrence quantification analysis. Perhaps not surprisingly, research found that the fairly implausible hypothesis that Bach’s brain was a Markov chain can be consistently rejected with sufficiently elaborate surrogates.

Apart from the above-reviewed examples that showcase how different aspects of musical arts can be studied by means of networks and physics, research along similar lines includes the application of complexity–entropy causality planes to distinguish songs [84], the identification of universal patterns in sound amplitudes of songs and music genres [85], the extraction of musical rhythmic patterns by means of community relevance in networks [86], and the quantification of soundscape dynamics of human agglomeration [87]. Paired with fundamental work on musical theory, for example concerning the geometry of musical chords [88], the hope is that such interdisciplinary research can contribute relevantly to the better understanding of music and its popularity across genres, geographical regions and time [89].

## 5. Literary arts

The digitalization of art had perhaps the biggest impact on literary arts. What previously existed only on paper became available as bits and bytes. Sites like the Google n-gram viewer at books.google.com/ngrams [28] and the Project Gutenberg at gutenberg.org, as well as social networking sites such as Twitter and Facebook, strongly facilitated quantitative research inquiries into written text on massive scales [90–101]. However, the bulk of this research was concerned primarily with statistical properties, or with finding memes or with similarities or differences in the texts, rather than with the content in terms of art.

**Figure 8.**
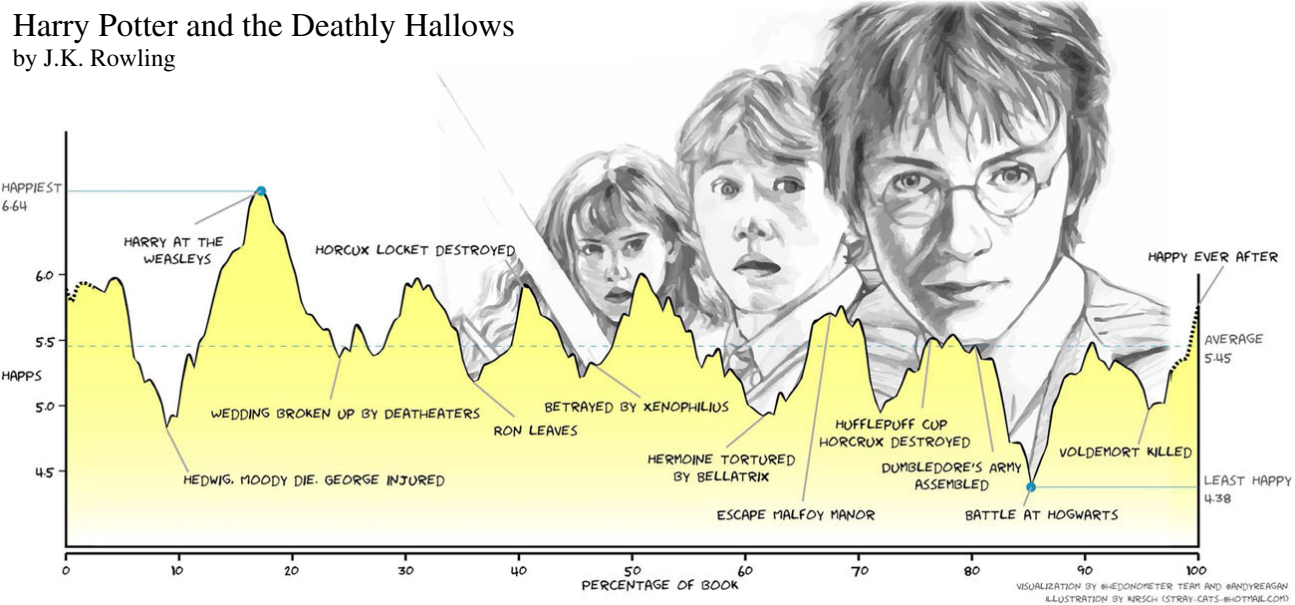
The emotional arc of *Harry Potter and the Deathly Hallows*, as determined by Reagan *et al.* [102], capturing the major highs and lows of the story. To generate such emotional arcs, authors analyse the sentiment of 10 000 word windows, which were slid through the text. The emotional content of each window was then evaluated using hedonometer.org with the labMT dataset [105,106]. The hedonometer.org webpage also provides interactive visualizations of emotional arc for many other books, stories, movie scripts, as well as for Twitter. Reproduced from [102].

One fascinating research effort that did focus on content is due to Reagan *et al.* [102], who studied the emotional arcs for a filtered subset of 1327 stories from Project Gutenberg’s fiction collection. As the main three independent methods of analysis, the authors used singular value decomposition—a standard linear algebra technique, Ward’s method to generate a hierarchical clustering of stories, which proceeds by minimizing the variance between clusters of books [103], and the self-organizing map [104]—an unsupervised machine learning method to cluster emotional arcs. Emotional arcs were constructed by analysing the sentiment of sliding 10 000 word windows using hedonometer.org and the labMT dataset [105,106]. The outcome for the J. K. Rowling’s *Harry Potter and the Deathly Hallows* is shown in figure 8, where the major highs and lows of the story are clearly inferable. As the authors note in their paper, the entire seven-book series can be classified as a ‘Kill the monster’ plot [107], while the many subplots and connections between them complicate the emotional arc of each individual book. The method, however, does not pick up emotional moments that are only discussed briefly, such as in a single paragraph or in a sentence. Whether the result shown in figure 8 corresponds with a reader’s experience depends on many factors, but being proud owners of all the books and DVDs of the *Harry Potter* series in our family, it does seem to fit rather well. My teenage daughter Ela and her friends agree that the happiest should be the ‘happy ever after’ rather than the ‘Harry at the Weasleys’, but that is coming from a single group of people. Others may feel differently and agree fully with what is shown in figure 8.

In the grander picture, the main finding of the Reagan *et al.* [102] paper is that the emotional arcs of all the stories included in their research are dominated by no more than six basic shapes. These are ‘Rags to riches’ (rise), ‘Tragedy’ or ‘Riches to rags’ (fall), ‘Man in a hole’ (fall-rise), ‘Icarus’ (rise-fall), ‘Cinderella’ (rise-fall-rise) and ‘Oedipus’ (fall-rise-fall). Importantly, the same six emotional arcs are obtained from all possible arcs by observing them as the result of three independent methods mentioned earlier, each with its own strength. Namely, the singular value decomposition finds the underlying basis of all of the emotional arcs, the clustering classifies the emotional arcs into distinct groups, and the self-organizing map generates arcs from noise which are similar to those in the corpus using a stochastic process. The result is thus truly robust and thoroughly supported by the presented evidence.

On a much smaller scale, Markovič *et al.* [108] recently studied the structure and complexity of texts in Slovene belles-lettres, with an emphasis on evaluating the differences in the texts for different age groups. Research revealed that the syntactic connectivity of words forms complex and heterogeneous networks that are characterized by an efficient transfer of information. It was also shown that with the increasing recommended age of readers, the length of texts, the average length of words, different combinations of phrases, and the complexity of social interactions between literary characters all increase. Conversely, the fraction of unique words was shown to decrease.

**Figure 9.**
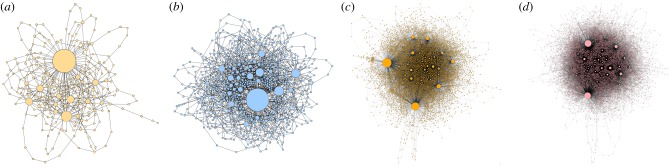
Language networks of selected Slovene belles-lettres for different age groups. Network A is for children age 1–5, network B is for children age 6–8, network C is for children age 9–11 and network D is for children age 12–14. Despite clearly visible differences in terms of the size and complexity of the networks, it is quite remarkable that the degree distribution is in all cases a power law. The average degree and the average clustering coefficient, however, increase with the recommended age of readers, while the modularity decreases. The average shortest path length decreases with increasing age, despite the increase in network size, which in turn gives rise to small-world properties [109]. Despite the changes in the average connectivity, the average shortest path length, and in the network size, the diameter of the networks is virtually identical in all age groups. For detailed data concerning these network properties, we refer to the original work. Reproduced from [108].

In terms of the language networks, as shown in figure 9, Markovič *et al.* [108] found that despite noticeable differences in network sizes, the degree distribution is a power law for all four age groups, and this with similar exponents. Apparently, some properties of syntactic patterns do not differ much for different age groups, although the relatively small sizes of the investigated networks did not allow a more precise comparison. Noteworthy, the observed scale-free property of language networks has been previously reported in several other works [110]. Further in terms of network properties, research revealed that the average degree and the average clustering coefficient increase with the recommended age of readers. This result goes in parallel with the decreasing tendency of unique word density. Namely, for higher age groups proportionally less unique words are used for longer texts and hence more word combinations are possible and indeed present, which in turn leads to more connections between individual words as well as to higher levels of cliquishness. For the same reason, the networks also become less modular with the recommended age of readers. On account of the increasing number of connections, the average shortest path length progressively decreases with increasing recommended age, despite the huge increase in network size. This, in turn, gives rise to the small-world topological features of the extracted syntactic networks. Interestingly, despite changes in the average connectivity, the average shortest path length and network size, the diameter of the network remains largely unchanged across age groups. Taken together, network science enabled an in-depth theoretical exploration of Slovene belles-lettres, with clear distinctions in statistical properties between different age groups, thus bridging art and exact sciences in a mutually rewarding way.

In moving the subject further ahead, Ferraz de Arruda *et al.* [111] recently noted that, while well-established word-adjacency or co-occurrence networks successfully grasp syntactical features of written texts, they are unable to capture topical structure. To remedy this, they have proposed a network model wherein adjacent paragraphs act as nodes, which are then connected whenever they share a minimal semantic content. As an example, Lewis Carroll’s *Alice’s Adventures in Wonderland* has been studied, and research showed that such an approach can reveal many semantic traits of a text that would remain hidden otherwise. Moving away from ‘just words’ and phrases to different degrees of mesoscopic structure, such as sentences, paragraphs, or chapters, could be the next step in bringing literary art closer still to methods of network science and physics.

## 6. Discussion

We have reviewed recent research that aims to bridge the gap between different artistic expressions and network science and physics. Although most of the works that we have covered are not about beauty as such, in retrospect, they do allow us to quantify and to understand what it is we find appealing or beautiful when we are subjected to a particular art form.

When it is food that we like, we can now appreciate which are the food pairings that make us take that extra bite, and which are the bridges between ingredients that make us revisit a restaurant. In broad terms, East Asian cuisine is our jam when neither food-pairing nor food-bridging should be on the menu, while Southeastern Asian cuisine appeals to those who do not like food-pairing but do like food-bridging. Western cuisine is all about food-pairing with almost no food-bridging, while Latin American cuisine should work well for those that like both food-pairing and food-bridging.

When we see a painting that we find beautiful, we can link this beauty to entropy and complexity. If we like ordered and repeating patterns, it is low entropy and high complexity, while everything ‘malerisch’ is high entropy and low complexity. The two physics quantities can be linked nicely to traditional concept in art history. Images formed by distinct and outlined parts yield many repetitions of a few ordinal patterns, and consequently, linear/haptic artworks are described by small values of entropy and large values of complexity. On the other hand, images composed of interrelated parts delimited by smudged edges produce more random patterns, and accordingly, painterly/optic artworks are expected to yield larger values of entropy and smaller values of complexity. It is possible to go even further and argue that Wölfflin’s dual concept of linear versus painterly and Riegl’s dichotomy of haptic versus optic are actually limiting forms of representation that demarcate the scale of all possibilities, and that in this regard the continuum of entropy and complexity values may help art historians to grade this scale more finely.

When we hear a song we then put on repeat, research at the interface of physics and network science can help us make out some of its properties that go beyond, or are outside the realm of, classic musical theory. Rhythm-based features from a network perspective, communities of different composers and genres, as well as complexity–entropy causality planes can all help us pinpoint what it is that we find beautiful in a particular song, improve personalized music recommendations, and improve also our understanding of what others around us find appealing in a particular piece of music. These approaches can also be used to try and forecast the future of several prominent artists and to decipher the growth dynamics of the music network. Not to take anything away from classic musical theory and the geometry of musical chords and similar concepts, the point we are trying to make is simply that network science and physics can play a very useful role too.

Lastly, when we read a story that really moves us, we can look up whether its emotional arc is something like a ‘Cinderella’ rise-fall-rise, or perhaps just an ‘Icarus’ rise-fall. We can also appreciate whether we prefer many entangled characters that are almost hard to keep track of, or just two or three main characters that drive the story. A network science perspective will also give us insights about modularity of the story, that is whether some parts of it are particularly removed from other parts, and whether they come together at some point or not. Here, the concept of mesoscopic text structures, such as sentences, paragraphs or chapters, could be the new frontier in quantitative linguistics, not just in terms of statistics but also in terms of content and storytelling and emotional arcs.

The underlying movement of people, knowledge, craftsmanship and techniques that enabled the evolution of the arts reviewed above has, in a grander perspective, to do with our cultural history. In this regard, too methods of network science and physics have played an important role in recent years to advance the subject [26–28]. The culturomics paper by Michel *et al.* [28], the bravely titled Quantitative analysis of culture using millions of digitized books, was perhaps even too optimistic in what precisely we might be able to learn from such a ‘big data’ exploration of our culture. Later online comments and research were quite quick to point out the limits to inferences of socio-cultural and linguistic evolution based on such data [112], which consequently also meant that quite a few research efforts, such as [113,114] for example, might have been too quick to jump on the culturomics bandwagon [115]. But this is often the nature of a fast moving and growing field. The initial over-enthusiasm and positivity are dampened by more measured and careful approaches latter on, until eventually a maturity phase sets in, to be followed by the inevitable decline.

Nonetheless, the popularity of this line of research, together with connections to artificial intelligence via computational aesthetics [22], has been growing steadily in recent years, touching also upon science production [116–118]—see matjazperc.com/aps—and peer review [119] to complement research on many other aspects of our societies that we have mentioned already in the introduction, including art as reviewed here. We hope this review will be informative and useful to researchers that are working at the fascinating interfaces of inherently different but also complementary research fields, and in particular to researchers that are seeking a way forward in mutually rewarding synergies between the arts and quantitative sciences.

## Data accessibility

Data sharing not applicable to this paper as no datasets were generated or analysed during the current study.
